# Identification of a Neutralizing Epitope on TOSV Gn Glycoprotein

**DOI:** 10.3390/vaccines9080924

**Published:** 2021-08-19

**Authors:** Claudia Gandolfo, Shibily Prathyumn, Chiara Terrosi, Gabriele Anichini, Gianni Gori Savellini, Davide Corti, Luisa Bracci, Antonio Lanzavecchia, Gleyder Roman-Sosa, Maria Grazia Cusi

**Affiliations:** 1 Virology Unit, Department of Medical Biotechnologies, University of Siena, 53100 Siena, Italy; claudia.gandolfo@unisi.it (C.G.); shibily.prathyumn@student.unisi.it (S.P.); chiara.terrosi@unisi.it (C.T.); gabriele.anichini@student.unisi.it (G.A.); gianni.gori@unisi.it (G.G.S.); 2 Institute for Research in Biomedicine, 6501 Bellinzona, Switzerland; davide.corti@humabs.ch (D.C.); alanzavecchia@vir.bio (A.L.); 3 Biochemistry Unit, Department of Medical Biotechnologies, University of Siena, 53100 Siena, Italy; luisa.bracci@unisi.it; 4 Structural Virology Unit, Virology Department, Institut Pasteur, 25-28 rue du Dr Roux, 75015 Paris, France; gleyder.roman-sosa@vetmed.uni-giessen.de

**Keywords:** Toscana virus, glycoprotein, epitope, vaccine

## Abstract

Emerging and re-emerging viral infections have been an important public health problem in recent years. We focused our attention on Toscana virus (TOSV), an emergent neurotropic negative-strand RNA virus of the *Phenuiviridae* family. The mechanisms of protection against phlebovirus natural infection are not known; however, it is supposed that a virus-neutralizing antibody response against viral glycoproteins would be useful to block the first stages of infection. By using an improved memory B cell immortalization method, we obtained a panel of human mAbs which reacted with TOSV antigens. We identified three epitopes of TOSV Gn glycoproteins by neutralizing mAbs using synthetic peptide arrays on membrane support (SPOT synthesis). These epitopes, separated in primary structure, might be exposed near one another as a conformational epitope in their native structure. In vivo studies were conducted to evaluate the humoral response elicited in mice immunized with the identified peptides. The results underlined the hypothesis that the first two peptides located in the NH_2_ terminus could form a conformational epitope, while the third, located near the transmembrane sequence in the carboxyl terminus, was necessary to strengthen neutralizing activity. Our results emphasize the importance of identifying neutralizing epitopes shared among the various phleboviruses, which could be exploited for the development of a potential epitope-based diagnostic assay or a polyvalent protective vaccine against different phleboviruses.

## 1. Introduction

Toscana virus (TOSV) belongs to the phlebovirus genus within the *Phenuiviridae* family under the order Bunyavirales [1]. TOSV was first isolated in 1971 in central Italy (in Monte Argentario, Grosseto Province) from two different species of sandflies, *Phlebotomus perniciosus* (*P. perniciosus*) and *Phlebotomus perfiliewi* (*P. perfiliewi*) [2,3,4]. TOSV is considered an emergent pathogen associated with acute neurological disease, such as meningitis, and occurs in Mediterranean countries during the summer months [5,6,7,8,9].

Despite the important role played by TOSV in central nervous system (CNS) infections, it remains a neglected agent and is rarely considered by physicians [10]. Like all members of the genus *Phlebovirus*, TOSV contains a segmented negative-strand RNA genome consisting of three non-covalently closed circular RNA segments that code, respectively, for the large protein (L) [11], the envelope glycoproteins (Gn and Gc) plus a non-structural protein (NSm) [12,13], and the nucleocapsid protein (N) together with the non-structural protein (NSs) [14].

The mechanisms of protection against natural infection of *Phlebovirus* are not known; however, it is supposed that a neutralizing antibody response against the viral glycoproteins could be necessary to block the first stages of infection. In several members of the family, such as Rift Valley fever virus (RVFV), La Crosse virus (LACV), Hantaan virus (HTNV), and TOSV, this neutralizing activity is a property of anti Gn-Gc antibodies [15,16,17,18,19,20,21]. In TOSV, however, a partial neutralizing activity was also shown for anti-N antibodies [22].

In this study, we focused our attention on TOSV Gn glycoprotein, since studies on the antigenicity of glycoproteins could be helpful to design epitopes that can be exploited as potential targets for the production of epitope-based diagnostics and vaccines against TOSV and other related *Phleboviruses*. In particular, we tried to identify viral Gn epitopes by using specific neutralizing antibodies. By immortalization of human memory B cells of a seropositive subject and parallel high-throughput screening for TOSV antigens, we localized three neutralizing epitopes. Two of these epitopes (Region 1 and Region 2) were located in the amino-terminal half of the Gn protein, while the third (Region 3) was close to the transmembrane region. To evaluate their immunogenicity, different combinations of these peptides were used for in vivo studies. All sera of immunized mice elicited, to a different extent, a good antibody response against Gn, laying the basis for the development of a polyvalent protective vaccine against different phleboviruses.

## 2. Materials and Methods

### 2.1. Viruses and Cells

Vero cells (ATCC CCL-81) were grown as a monolayer in Dulbecco’s modified Eagle’s medium (DMEM) (Lonza, Milan, Italy) supplemented with 5% heat-inactivated fetal calf serum (FCS) (Lonza) and 100 U/mL penicillin-streptomycin (HyClone Europe, Milan, Italy) at 37 °C. Human embryonic kidney (HEK)-Lenti-X 293 cells (Clontech, Milan, Italy) were grown as a monolayer in DMEM (Lonza, Milan, Italy) supplemented with 10% heat-inactivated FCS (Lonza) and 100 U/mL penicillin-streptomycin at 37 °C. Spodoptera frugiperda cells (Sf9) were propagated in SF-900II medium (GIBCO InVitrogen, Milan, ITALY) containing streptomycin (100 mg/mL; Life Technologies), and penicillin (100 UI/mL; Life Technologies).

Toscana virus (TOSV) strain 1812 cultured on Vero cells (isolated from a clinical specimen S. Maria alle Scotte Hospital, Siena, Italy) was plaque purified and propagated for viral stock preparation. Sandfly fever Naples virus (SFNV strain Sabin, kindly provided by ISS), sandfly fever Sicilian virus (SFSV, strain Sabin, provided by ISS), and Punique virus (PUNV, strain P1_B4_2008, isolated from a sandfly pool collected in Tunisia) were grown on Vero cells at the virology laboratory of the University of Siena. Purified TOSV for ELISA screening was prepared as described elsewhere [23].

### 2.2. Human PBMC Isolation

Peripheral blood mononuclear cells (PBMCs) were isolated from human blood, diluted in an equal volume of phosphate buffered saline (PBS) and further separated by Ficoll-Hypaque gradient by centrifugation at 1800 rpm for 20 min at RT. Cells were collected from the plasma/Ficoll interface, resuspended in PBS and centrifuged at 1300 rpm for 10 min at RT. Cells were finally resuspended in RPMI 1640 medium (Hyclone, Cramligton, UK) and counted. This research was carried out according to the principles of Helsinki declaration. Ethical approval was obtained from the local ethical committee for clinical trials (authorization TOSV2016_19/04/2016) (Comitato Etico Regione Toscana-Area Vasta Sud Est) in terms of General Data Protection and Regulation (GDPR) upon written informed consent signed by all subjects prior to participating in this study [24,25].

### 2.3. Immortalization of Human Memory B Cells

PBMCs and plasma samples were obtained from a patient with meningitis, infected by TOSV, during the acute phase and one year after the infection. At the Institute for Research in Biomedicine (Bellinzona Switzerland), frozen PBMCs were thawed and immortalized using EBV in the presence of CpG, as previously described [26]. Briefly, IgG^+^ memory B cells were isolated using magnetic cell separators (MACS) and negative and positive selection kits (Miltenyi Biotec, Bergisch Gladbach, Germany). Then, memory B cells were seeded at 10 or 50 cells per well in 96 U-bottom microplates (Greiner, ThermoFisher Scientific) in complete medium containing 2.5 μg/mL CpG 2006, in the presence of EBV (30% supernatant of B95-8 cells) and irradiated allogeneic mononuclear cells (50,000 per well). After 2 weeks, the culture supernatants were screened for specific antibodies by Intellicyt Cytometer against TOSV-infected VERO cells. Specific TOSV antibodies were purified from culture supernatants by affinity chromatography on protein A columns (Amersham) and stored at −20 °C for further testing.

### 2.4. SPOT Peptide Synthesis

A set of 82 overlapping peptides of 15 aa, shifted by five residues, spanning aa 1–416 of TOSV Gn glycoprotein (GenBank accession no: AEM36009.1 from aa 308–834) was synthesized as an array on derivatized cellulose based membranes using SPOT technology [27,28]. In the sequence, the cysteines were replaced by serine. The membrane immobilized peptides were first saturated in MBS (pH 7.0) containing 2% non-fat milk and 0.05% Tween 20 in Tris Buffered Saline (TBS) o/n at 4°C. After three washing steps with TBS containing 0.05% Tween 20 (TTBS), peptides were probed with each human mAb at a concentration of 5 μg/mL in MBS for 90 min at 37 °C. Following the other three washing steps, the membrane was incubated with HRP-conjugated goat anti-mouse IgG (Merk Life Science S.r.l., Milano, Italy) at 1:8000 dilution and, after three washing steps, an enhanced chemiluminescent substrate kit was used for detection of HRP development (Thermo SCIENTIFIC, Monza, Italy) according to the manufacturer’s instructions. The spots were evaluated with a gray scale of intensity after image acquisition using ImageQuantTL, as described [29].

### 2.5. Expression and Purification of Recombinant Proteins and Peptides

**Figure 1:**
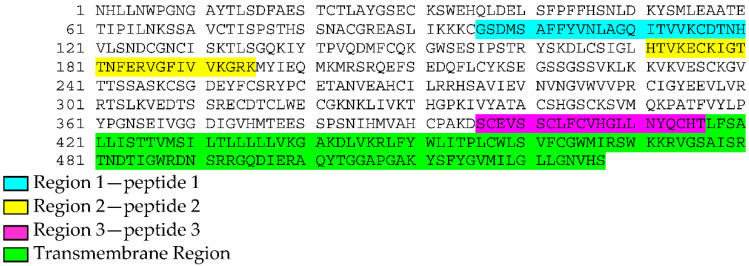
Three aminoacid sequences identified by pepscan analysis.

Plasmid vector pGex-2T (Amersham Biosciences, Milano, Italy) was used for the construction of a plasmid-containing defined peptide sequence of TOSV Gn glycoprotein. The gene sequence, containing peptides 1 and 2 (Figure 1), was amplified from the purified viral RNA by reverse transcriptase PCR (RT-PCR) using specific primers. The reaction was carried out using the Super Script III one-step RT-PCR mix (Invitrogen) by one cycle of reverse transcription at 50 °C for 30 min and 94 °C for 2 min, followed by 40 cycles of PCR (15 min at 94 °C, 30 s at 56 °C and 1 min at 68 °C) and 5 min at 68 °C. The primers used were:

Forward primer: 5′-AAGGATCCGGAAGTGATATGTCG-3′

Reverse primer: 5′-GGGGAATTCTCATCTTCATCTGCTC-3′

The amplified gene was then cloned into a pGex-2T vector and linearized with Bam HI and EcoRI. The plasmid was named GC683. Another plasmid, named GC727, containing peptide 1, 2 and 3 sequences of TOSV Gn glycoprotein was constructed by inserting the sequence coding for peptide 3 (Figure 1) in GC683 plasmid after linearization with EcoRI (Infusion cloning Takara Bio Inc, Kusatsu, Shiga Japan). A five-alanine coding sequence upstream of peptide 3 was fused with the linearized vector:

5′-GAG CAG ATG AAG ATG AGA ATT CAT GCG GCGGCGGCGGCG TCC TGT GAG GTT AGC AGC TGC CTA TTC TGT GTG CAC GGA CTG CTT AAC TAC CAG TGC CAC ACC TGA CTC GAG GAA TTC ATC GTG ACT GAC TGA CGA-3′.

The top 10 competent cells were transformed with GC683 and GC727 plasmids, and protein expression was induced by 1mM IPTG (Thermo Fisher Scientific (Ultrospec 2100 pro, Amersham Biosciences, Milano, Italy). Cultures were allowed to grow for 3 h at 37 °C, and cells were harvested by centrifugation at 4500 rpm for 20 min. The expressed recombinant proteins, pep GC683 and pep GC727, were purified from the cells as previously described [30]. The purified proteins were then quantified by Bradford reagent (Avantor, Leuven, Belgium) at 595 nm. SDS PAGE was then performed to evaluate the purity of the protein by staining the gel with Coomassie Brilliant Blue (CBB) (Bio-rad, Milano, Italy). The specificity of the protein was confirmed by Western blot analysis (data not shown). The protein was then stored at −80 °C until further use. Assessment of endotoxin concentration (<0.5 endotoxin units/mL) during the protein purification process was performed by using the ToxinSensor Chromogenic LAL Endotoxin Assay Kit (GeneScript, Leiden, Holland) according to the the manufacturer’s instructions. The construct encoding the ectodomain fragment (Arg307-Thr725) of TOSV Gn protein from the strain Sotkamo (AMY16460.1) with a carboxyl terminal double Strep-Tag was generated by PCR (Forward primer: 5′-GACGATGACGATAAGGCCGGTTGG-3′ and Reverse primer: 5′-GGTGTGGCACTGGTAGTTCAGCAGGCC-3′) and followed by religation of the PCR product. The template used was a synthetic gene that encodes the full-length glycoprotein precursor with a double Strep-Tag at the carboxyl terminus already cloned in the pMT/V5-His A plasmid (Invitrogen, Monza, Italy) for expression in Drosophila S2 cells. The recombinant plasmid was co-transfected with the puromycin resistance gene-expressing plasmid pCoPuro in Drosophila S2 cells at a ratio of 1:20, and stable cell lines were established in the presence of puromycin (7 μg mL^−1^). The protein was purified from the cell supernatants as previously described [31] and kept at −80 °C until use.

### 2.6. Mice Immunization

Four-week-old female BALB/c mice (Charles River, Milan, Italy) were used in the immunization experiments. Each experiment was repeated three times to assess the reproducibility of results. Twelve groups of five mice each were immunized with 100μg/mice of Gn peptides in different combinations every 2 weeks by four intraperitoneal (IP) injections (Table 1). All animal experiments were approved by the local ethics committee and carried out in strict compliance with the Institutional Animal Care and Use Committee (IACUC) guidelines and in accordance with the 2010/63/EUDirective (http://eurlex.europa.eu/LexUriServ/LexUriSev.do?uri=OJ:L:2010:276:0033:0079:EN:PDF, accessed on 22 September 2010) of the European Parliament and the Council for the Protection of Animals Used for Scientific Purposes. Peptide 1, 2 and 3 as single units conjugated with the KLH carrier were purchased from Peptide Facility (CRIBI—Centro di Biotecnologie Università di Padova). All the combinations of antigens contained an equal volume of Montanide (50 µL/mouse) as adjuvant.

Two weeks after the last immunization, all mice were sacrificed, blood was drawn, and serum was collected and stored at −20 °C for further analysis.

### 2.7. ELISA

Microtiter plates (Labsystem, Helsinki, Finland) were coated with 100 μL per well of either purified whole TOSV or purified Gn glycoprotein protein (1 μg/mL conc.) in 0.1 M carbonate buffer (pH 9.6) and incubated o/n at 4 °C. To prevent non-specific binding, 100 μL of non-fat dry milk (0.5%) was added to each well for 1 h at RT. After three washing steps with PBS containing 0.05% Brij, 100 μL of mice sera or human mAbs diluted 1:50 in dilution buffer (PBS + 0.05% Brij + 10% FBS) were added to each well and the plates were incubated at 37 °C for one hour. Post incubation, the plates were washed and 100 μL of peroxidase-conjugated anti-mouse IgG or anti-human IgG (Sigma-Aldrich) were added followed by incubation at 37 °C for one hour in dark. The plates were then washed prior to adding 100 μL of 3,3′,5,5′-tetramethylbenzidine to each well (TMB One Component HRP Microwell Substrate, Tebu-bio laboratories) for 15 min. A negative control corresponding to a pool of TOSV seronegative human sera (previously screened in our lab) was included in the assay. The reaction was stopped by adding 100 μL of 1 N H_2_SO_4_ solution, and the plates were read immediately at 450 nm.

### 2.8. Neutralization Test

Virus neutralization was carried out on Vero cells in a 96 well microplate. Briefly, two-fold serial dilutions (50 μL) of immunized mice serum or human mAbs were added to an equal volume of TOSV containing 100 TCID_50_ and incubated for 90 min at 37 °C. Fifty μL of cells (10^6^/mL) were suspended in Minimum Essential Media (MEM, InVitrogen, Milan, Italy) with 5% FCS and added to each well. Five days after incubation at 37 °C, the cultures were examined microscopically for the presence of a cytopathic effect. The 50% end point titer of the neutralizing serum was calculated using the Reed and Muench method [32]. A titer <1/4 was considered negative. The same protocol was used for the neutralization assay versus SFNV, SFSV and PUNV.

### 2.9. Indirect Immunofluorescence Assay

Sf9 cells were infected with baculovirus expressing the ectodomains of TOSV glycoproteins (Bac-Gn and Bac-Gc) as described elsewhere [22]. Lenti-X HEK293 were transfected with 1µg of Gn-expressing plasmid using the GeneJuice reagent (Merck-Millipore, Milan, Italy), as suggested by the manufacturer. Vero cells were infected with TOSV 1812. All these cells were spotted on slides and fixed for 10 min at room temperature with cold methanol/acetone and screened by indirect immunofluorescence (IFA).

Diluted mice sera (1:50 in PBS) or human mAbs were added to the slides, followed by incubation at 37 °C for 30 min. Subsequently, the slides were washed with PBS for 5 min, and FITC-conjugated anti-mouse or anti-human secondary antibody (Sigma, Milan, Italy) was added to the slides and incubated for 20 min at 37 °C. After the final wash, immunofluorescence was visualized using a Diaplan microscope (Leica Microsystems, Milan, Italy). The cross-reactivity of mouse sera against Sicilian virus (SFSV), Naples virus (SFNV), and Cyprus Virus (CYPR) was tested using a commercially available kit “sandfly fever virus Mosaic 1 types: Sicilian, Naples, Toscana, Cyprus IFA” (EUROPattern) following the manufacturer’s instructions.

### 2.10. Statistical Analysis

The statistical analysis of differences between means was performed using Stat View statistical software (Abacus Concepts, Berkeley, CA, USA). Neutralization titers were presented as geometric mean ± standard deviation. *p* < 0.05 was considered significant.

## 3. Results

### 3.1. Isolation and Screening of a Panel of TOSV Human Monoclonal Antibodies

**Figure 2:**
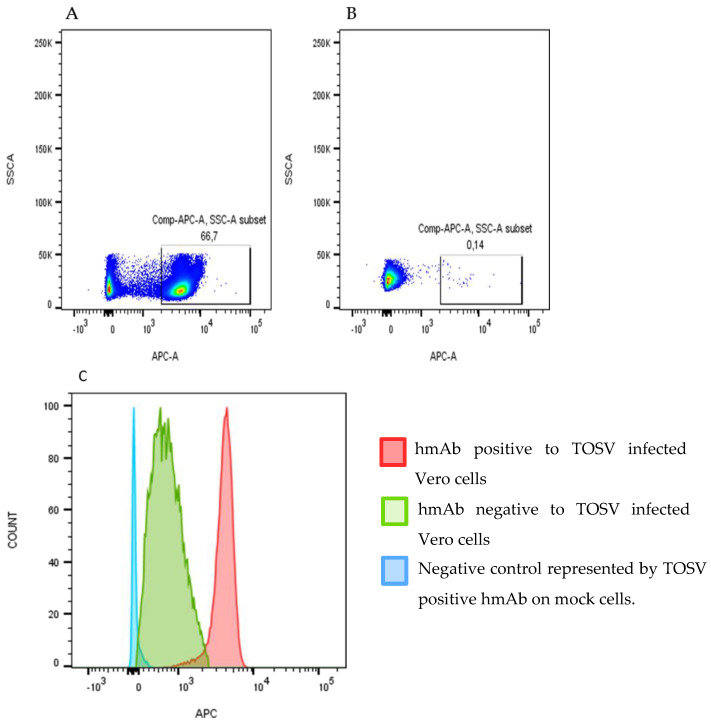
High-throughput FACS analysis using TOSV-infected VERO cells. (**A**) shows the identification of TOSV-positive human mAb by using an anti-hFc-APC secondary antibody (BD Biosciences) reacted with infected cells, while (**B**) shows a TOSV-negative hmAb. (**C**) shows the difference between the fluorescence of a positive and a negative hmAb to TOSV-infected Vero cells versus mock cells.

PBMCs and serum were collected from a patient infected by TOSV during the acute phase and one year later. After immortalization, B cell culture supernatants containing Ig were screened by high-throughput FACS analysis using TOSV infected VERO cells (Figure 2).

Based on this primary screening, 112 mAbs which were positively reactive against TOSV infected cells were selected. Among them, 7 antibodies were derived from PBMCs of the acute phase (identified as TVA mAbs), while 105 were from PBMCs collected one year post infection (TVB mAbs). The whole mAbs panel was first screened against recombinant TOSV N protein by ELISA in order to exclude those positive to the N protein.

Fifty-eight mAbs (5 from the acute phase and 53 from the convalescent phase) were then analyzed by immunofluorescence with Sf9 cells infected with recombinant baculoviruses, Bac-Gn or Bac-Gc, expressing the ectodomains of TOSV Gn and Gc proteins, respectively. Thirteen out of 58 mAbs were positive to Sf9-Gn and five showed positivity to Sf9-Gc; 40 tested negative to both the proteins. The 58 mAbs were also screened by neutralization assay with a TOSV strain 1812. We did not find any neutralizing activity among the monoclonal antibodies derived from the acute phase sample, although the human serum of the acute phase had a neutralizing titer of 1/161. However, twenty mAbs obtained from the convalescent phase sample had a high neutralization activity with titer values ranging from 1/128 to >1/512 (Table 2). These neutralizing mAbs were analyzed by immunofluorescence with Lenti-X HEK293 cells transfected with plasmids expressing the whole glycoproteins. Our attention was first addressed to the Gn glycoprotein, because all the mAbs with a high neutralization titer were positive to the Gn glycoprotein. Twelve mAbs, previously negative by IFA on Sf9-Gn cells and lacking the transmembrane region, were positive on Lenti-X HEK293-Gn, expressing the whole protein and suggesting that these antibodies recognized a region close to the transmembrane domain (data not shown).

### 3.2. Identification of Neutralizing TOSV Gn Epitopes by Pepscan Analysis

SPOT peptide synthesis [27,28] was used to generate membranes of 82 spots of 15 aa–long peptides shifted by 5 residues, covering TOSV Gn glycoprotein from aa 1 to aa 416 (GenBank accession no: AEM36009.1 from aa 308–834). These membranes were used to identify Gn epitopes recognized by previously selected neutralizing mAbs. We also included three aspecific mAbs as negative controls.

Most of the mAbs, in particular those with a neutralizing activity >1/128, reacted with the same strings of Gn peptides. Two of these linear peptides were localized in the amino-terminal half of the protein; one was instead close to the transmembrane region of the glycoprotein (Figure 1). The three regions identified by the neutralizing mAbs were also recognized by the sera of the acute as well as the convalescent phase. Furthermore, the serum drawn during the convalescent phase showed a higher reactivity against the spotted peptides, compared with the serum of the acute phase.

The three regions identified by pepscan analysis were as follows: the first two regions, 1 and 2, were localized in the amino-terminal of the Gn glycoprotein, while region 3 was located near the transmembrane sequence.

### 3.3. Cross-Reactivity of Human mAbs

Since cross-reactivity of sera among phleboviruses is quite common, we analyzed the ability of some mAbs to neutralize viruses of the phlebovirus genus, such as sandfly fever Naples virus (SFNV), sandfly fever Sicilian virus (SFSV), and Punique virus (PUNV). A preliminary screening detected one anti-TOSV mAb that neutralizes SFNV. Unfortunately, because of the small amount of purified mAbs, we could not test them against other phleboviruses. On the contrary, none of the tested mAbs against SFSV and PUNV showed neutralizing activity (Table 3).

### 3.4. Immunization of Mice with Peptides

**Figure 3:**
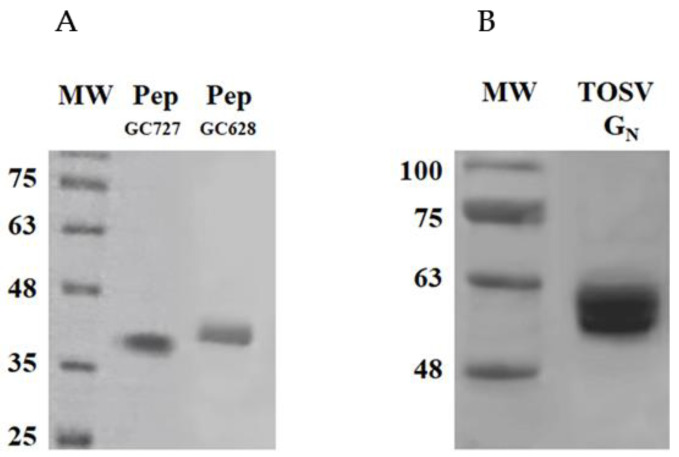
SDS_PAGE of TOSV recombinant proteins. (**A**) 5 µg of purified pep GC683 and GC727 and (**B**) TOSV Gn protein were analyzed on a 12% polyacrylamide gel under reducing conditions and stained with Coomassie Blue. MW: SharpmassVI, Euroclone.

Different combinations of these peptides were used for in vivo studies to determine their immunogenicity. The peptides were used as a single antigen linked with KLH as carrier, or in combination, or fused with GST as carrier protein. Peptides 1 and 2 were expressed by a plasmid named GC683, whereas peptides 1, 2 and 3 were expressed by a plasmid named GC727 as described in Materials and Methods (Figure 3).

Four weeks-old female Balb/c mice were immunized intraperitoneally as described in Table 1. Four consecutive immunizations of 5 mice/group were carried out at two weeks interval.

The antibody response to the different combinations of peptides was evaluated in mice. After four cycles of immunizations, mice were sacrificed, and the sera obtained from each group were tested for the presence of antibodies against both Gn protein and purified TOSV by ELISA.

**Figure 4:**
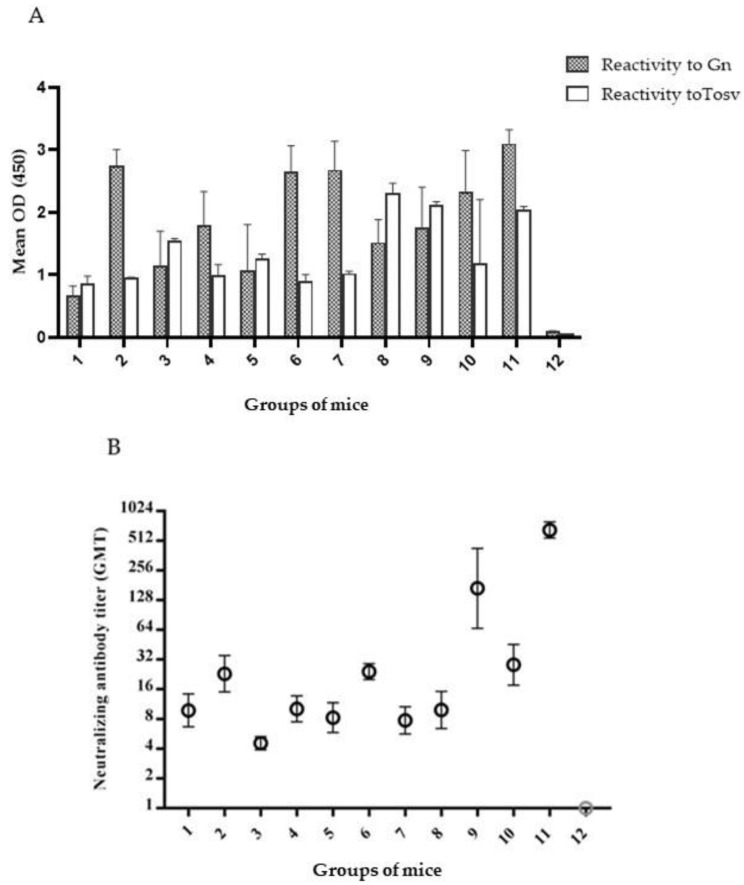
(**A**) Evaluation of the humoral response of immunized mice with different combinations of antigens, either against rGn or whole TOSV, by ELISA. (**B**) Evaluation of the neutralizing antibody response in immunized mice measured in quadruplicate. Results are reported as geometric mean titer (GMT, black circles) with 95% confidence interval (95% CI). **Group 1**: peptide 1-KLH; **Group 2**: peptide 2-KLH; **Group 3**: peptide 3-KLH; **Group 4**: peptide 1-KLH and peptide 2-KLH; **Group 5**: peptide 1-KLH and peptide 3-KLH; **Group 6**: peptide 2-KLH and peptide 3-KLH; **Group 7**: peptide 1-KLH, peptide 2-KLH and peptide 3-KLH; **Group 8**: pep GC683; **Group 9**: pep GC683 and peptide 3-KLH; **Group 10**: pep GC727; **Group 11**: rGn; **Group 12**: PBS.

All the mice elicited, to a different extent, an antibody response against all combinations of the peptides. ELISA results, using Gn as antigen, showed that sera of mice immunized with single synthetic peptides or combinations of them were all reactive. The group immunized with Pep1-KLH developed a low level of antibodies, while those immunized with Pep 2-KLH and Pep 3-KLH or other combinations containing peptides 2 and 3 were able to induce a high level of antibodies reacting with Gn. Sera screened by using the purified whole TOSV as antigen showed positivity in all the immunized mice groups, although at varying levels. Mice immunized with Pep 3-KLH or combinations containing peptide 3 showed a good response. Moreover, the mice group immunized with Pep GC683 alone or with Peptide 3-KLH showed the best response (Figure 4A).

Mice sera were also tested by immunofluorescence on TOSV-infected Vero cells. Only the sera of mice immunized with Pep 2-KLH or Pep3-KLH did not react with the viral glycoprotein in immunofluorescence, indicating that it is possible that each single conformomer was not similar to that of the original protein (data not shown).

A neutralization assay was also performed to determine specific neutralizing activity against TOSV. The mice groups immunized with the single peptides 1, 2 and 3 showed low neutralization titers of 1/10, 1/23 and 1/5, respectively. Additionally, the groups immunized with different combinations of single peptides showed a modest neutralization titer (range 1/8–1/24), as did the mice immunized with Pep GC683 and Pep GC727 (titer of 1/10 and 1/28, respectively). On the contrary, the mice group immunized with a combination of Pep GC683 + Pep3-KLH showed a significantly higher titer value (1/183) (Figure 4B).

### 3.5. Serological Cross-Reactivity of Immunized Mice

The immunized mice sera were tested for cross-reactivity against some viruses of the Phlebovirus genus, including SFNV, SFSV, CYPR (Cyprus viruses) and PUNV using a commercially available IFA kit. The sera of all groups showed cross-reactivity with SFNV, but only the serum of mice immunized with Pep1-KLH+ Pep 2-KLH recognized SFSV and CYPR. PUNV reacted only with sera of groups receiving Pep 3-KLH alone or in combination, as an immunogen (Table 4).

## 4. Discussion

By using an improved memory B cell immortalization method [26], combined with parallel high-throughput screening for TOSV antigens, we isolated a panel of human mAbs which were characterized with regard to epitope specificity and neutralizing activity. We demonstrated that most of the antibodies were directed to TOSV Gn glycoprotein and that they acquired a high degree of magnitude of response with the maturation of the adaptative immune response; indeed, they were all obtained from PBMCs of the convalescent phase blood sample.

With pepscan analysis, we were able to identify three amino acid regions that were always recognized by the neutralizing mAbs. Two of these epitopes (Region 1 and Region 2) were located in the amino-terminal half of Gn protein, while the third (Region 3) was close to the transmembrane region. We first hypothesized that they were a part of a larger conformational epitope that could be recognized when exposed on the viral envelope in the natural structure of the protein.

To evaluate their immunogenicity, different combinations of these peptides were used for in vivo studies. All the sera of immunized mice were positive to Gn in the ELISA assay. This result demonstrated that the mice elicited, to different extents, a good antibody response against the viral glycoprotein. Likewise, the sera tested against the whole virus showed a high antibody level.

In particular, sera of mice immunized with Pep GC683 or Pep GC727 expressing peptides 1 and 2 or 1, 2, and 3 fused with GST, respectively, showed very good results once tested against the whole TOSV either in ELISA (Figure 4) or IFA (data not shown). This finding indicates that peptides 1 and 2 might represent a conformational structure similar to that of the original protein which is recognized by TOSV specific sera, although we cannot exclude that a linear epitope with a dominant sequence for peptide 3 could also be a favourable target.

Concerning the induction of neutralizing antibodies, all combinations of peptides induced a modest response with a range varying from 1/8 to 1/28, but Pep GC683 + Pep3-KLH induced a better response (GMT 1/183). Therefore, this result supports the hypothesis that Pep 1 and Pep 2 could be part of a conformational epitope capable of inducing neutralizing antibodies, and Pep 3, located near the transmembrane region, could strengthen this activity by hindering the binding of TOSV to the cell receptor. Indeed, Pep GC683 alone did not mount a high titer of neutralizing antibodies, but the addition of Pep 3-KLH enhanced the neutralizing response in vivo. Thus, it appears that both epitopes are involved in the neutralization of virus infectivity. In particular, they might act in synergy by reacting with distinct domains of the Gn glycoprotein and inducing a steric hindrance at the binding site with the receptor, a phenomenon already known to occur [33]. Thus, this binding could block the virus entry into the cell, mediated by the glycoproteins, and, consequently, inhibit viral replication. Moreover, considering the importance of identifying neutralizing epitopes shared by other phleboviruses, we evaluated the serological cross-reactivity of TOSV mAbs with neutralizing activity to other phleboviruses. Cross-reactivity is associated with linear as well as conformational epitopes [34]. The well-documented serological relationships among viruses of the same genus provides the rationale behind identifying conserved epitope regions on viral proteins [35] and their use for diagnostic as well as treatment purposes.

We found only one mAb (TVB 27) which neutralized SFNV, which has a Gn sharing 90% homology with TOSV Gn. This was only a preliminary screening, and a larger analysis must be carried out with the aim of detecting the epitopes shared by other phleboviruses that could be exploited for a potential vaccine against other sandfly viruses.

The sera of all groups of mice were also tested by immunofluorescence against SFNV, SFSV, PUNV and CYPR. All sera cross-reacted with SFNV; sera of mice immunized either with Peptide 3-KLH alone or its combinations reacted with Punique virus, which had 70% sequence similarity with Peptide 3. Unexpectedly, only the sera obtained from mice immunized with peptides 1-KLH + 2-KLH cross-reacted with SFNV, SFSV and CYPR, probably because this combination induced antibodies with a higher affinity than those elicited by single peptides.

Finally, it is worthy to note that these results were based only on the screening of mAbs derived from one patient. Thus, this study will be enlarged to other TOSV-positive patients to characterize further antibodies and extend the spectrum of functional epitopes. The results could be exploited for the development of potential epitope-based diagnostic assays or as the starting point for the development of a polyvalent vaccine protective against several sandfly viruses.

## Figures and Tables

**Table 1:** Mice immunization chart.

Group of Mice	Antigens Injected for Immunization
Group 1	Peptide 1-KLH
Group 2	Peptide 2-KLH
Group 3	Peptide 3-KLH
Group 4	Peptide 1-KLH and peptide 2-KLH
Group 5	Peptide 1-KLH and peptide 3-KLH
Group 6	Peptide 2-KLH and peptide 3-KLH
Group 7	Peptide 1-KLH, peptide 2-KLH and peptide 3-KLH
Group 8	Pep GC683
Group 9	Pep GC683 and peptide 3-KLH
Group 10	Pep GC727
Group 11	rGn
Group 12	PBS

**Table 2:** Human mAbs tested for neutralizing activity against TOSV.

**mAbs of Acute Phase**	
**mAbs**	**NT Titer**
5	neg
**mAbs of Convalescent Phase**	
**mAbs**	**NT Titer**
26	neg
7	<1/128
8	1/128–1/512
12	>1/512

No mAbs of the acute phase had a neutralizing activity, while twenty mAbs obtained from the convalescent phase sample had high neutralization activity with titer values ranging from 1/128 to >1/512.

**Table 3:** Neutralization assay of mAbs against SFNV, PUNV and SFSV.

Antibody ID	NT Titer			
	TOSV	SFNV	PUNV	SFSV
TVB 27	1/161	1/25	-	-
TVB 73	>1/512	-	-	-
TVB 147	1/645	-	-	-
TVB 161	1/645	-	-	-
TVB 164	1/6	-	-	-

**Table 4:** Cross-reactivity among mice sera and SFNV, SFSV, CYPR, PUNV Phleboviruses.

GROUPS	CROSS-REACTIVITY			
	SFNV	SFSV	CYPR	PUNV
**Group 1 (Pep 1-KLH)**	+	−	−	−
**Group 2 (Pep 2-KLH)**	+	−	−	−
**Group 3 (Pep 3-KLH)**	+	−	−	+
**Group 4 (Pep 1-KLH + Pep2-KLH)**	+	+	+	−
**Group 5 (Pep 1-KLH + Pep 3-KLH)**	+	−	−	+
**Group 6(Pep2-KLH + Pep3-KLH)**	+	−	−	+
**Group7(Pep1-KLH+Pep2-KLH + Pep3-KLH)**	+	−	−	+
**Group 8 (PepGc 683)**	+	−	−	−
**Group 9 (PepGc 683 + Pep3-KLH)**	+	−	−	+
**Group 10 (Pep Gc 727)**	+	−	−	+
**Group 11 (rGn)**	−	−	−	−
**Group 12 (PBS)**	−	−	−	−

## References

[1] Abudurexiti A., Adkins S., Alioto D., Alkhovsky S.V., Avšič-Županc T., Ballinger M.J., Bente D.A., Beer M., Bergeron É., Blair C.D. (2019). Taxonomy of the order *Bunyavirales*: Update 2019. Arch. Virol..

[2] Verani P., Ciufolini M.G., Nicoletti L., Balducci M., Sabatinelli G., Coluzzi M., Paci P., Amaducci L. (1982). Ecological and epidemiological studies of Toscana virus, anarbovirus isolated from Phlebotomus. Ann. Ist. Super. Sanita.

[3] Verani P., Nicoletti L., Ciufolini M.G. (1984). Antigenic and biological characterization of Toscana virus, a newPhlebotomus fever group virus isolated in Italy. Acta Virol..

[4] Verani P., Ciufolini M.G., Caciolli S., Renzi A., Nicoletti L., Sabatinelli G., Bartolozzi D., Volpi G., Amaducci L., Coluzzi M. (1988). Ecology of viruses isolated from sand flies in Italy and characterized of a new Phlebovirus (Arabia virus). Am. J. Trop. Med. Hyg..

[5] Valassina M., Cusi M.G., Valensin P.E. (2003). A Mediterranean arbovirus: The Toscana virus. J. NeuroVirology.

[6] Valassina M., Valentini M., Pugliese A., Valensin P.E., Cusi M.G. (2003). Serological survey of toscana virus infections in a high-risk population in Italy. Clin. Diagn. Lab. Immunol..

[7] Charrel Rèmi N., Pierre G., Josè-Maria N., Loredana N., Anna P., Paz S., Antonio T., de Lambelleire X.E. (2005). Emergence of Toscana Virus in Europe. Emerg. Infect. Diseas..

[8] Sanbonmatsu-Gàmez S., Pèrez-Ruiz M., Palop-Borràs B., Navarro-Marì J.M. (2009). Unusual Manifestation of Toscana virus Infection, Spain. Emerg. Infect. Dis..

[9] Cusi M.G., GoriSavellini G., Zanelli G. (2010). Toscana virus epidemiology: From Italy to beyond. Open Virol. J..

[10] Charrel R.N., Bichaud L., De Lamballerie X. (2012). Emergence of Toscana virus in the Mediterranean area. World J. Virol..

[11] Accardi L., Gro M.C., Di Bonito P., Giorgi C. (1993). Toscana virus genomic L segment: Molecular cloning, coding strategy and amino acid sequence in comparison with other negative strand RNA viruses. Virus Res..

[12] Di Bonito P., Mochi S., Gro M.C., Fortini D., Giorgi C. (1997). Organization of the M genomic segment of Toscana phlebovirus. J. Gen. Virol..

[13] Gro M.C., Di Bonito P., Fortini D., Mochi S., Giorgi C. (1997). Completion of molecular characterization of Toscana phlebovirus genome: Nucleotide sequence, coding strategy of M genomic segment and its aminoacid sequence comparison to other phleboviruses. Virus Res..

[14] Giorgi C., Accardi L., Nicoletti L., Gro M.C., Takehara K., Hilditch C., Morikawa S., Bishop D.H. (1991). Sequences and coding strategies of the SRNAs of Toscana and Rift Valley fever viruses compared to those of Punta Toro, Sicilian Sandfly fever, and Uukuniemi viruses. Virology.

[15] Saluzzo J.F., Anderson G.W., Hodgson L.A., Digoutte J.P., Smith J.F. (1989). Antigenic and biological properties of Rift Valley fever virus isolated during the 1987 Mauritanian epidemic. Res. Virol..

[16] Saluzzo J.F., Anderson G.W., Smith J.F., Fontenille D., Coulanges P. (1989). Biological and antigenic relationship between Rift Valley fever virus strains isolated in Egypt and Madagascar. Trans. R. Soc. Trop. Med. Hyg..

[17] Gonzalez-Scarano F., Shope R.E., Calisher C.E., Nathanson N. (1982). Characterization of monoclonal antibodies against Gn and N proteins of La Crosse and Tahyna, two California serogroup bunyaviruses. Virology.

[18] Grady L., Kinch W. (1985). Two monoclonal antibodies against La Crosse virus show host-dependent neutralizing activity. J. Gen. Virol..

[19] Arikawa J., Schmaljohn A.L., Dalrymple J.M., Schmaljohn C.S. (1989). Characterization of Hantaan virus envelope glycoprotein antigenic determinants defined by monoclonal antibodies. J. Gen. Virol..

[20] Di Bonito P., Bosco S., Mochi S., Accardi L., Ciufolini M.G., Nicoletti L., Giorgi C. (2002). Human antibody response to Toscana virus glycoproteins expressed by recombinant baculovirus. J. Med. Virol..

[21] Cusi M.G., Valentini M., Valensin P.E., Valassina M. Immune response to the neurotropic Toscana virus infection: Preliminary data. Proceedings of the 1st SIV International Workshop on Neurovirology.

[22] Cusi M.G., Valensin P.E., Donati M., Valassina M. (2001). Neutralization of Toscana virus is partially mediated by antibodies to the nucleocapsid protein. J. Med. Virol..

[23] Soldateschi D., dal Maso G.M., Valassina M., Santini L., Bianchi S., Cusi M.G. (1999). Laboratory diagnosis of Toscana virus infection by enzyme immunoassay with recombinant viral nucleoprotein. J. Clin. Microbiol..

[24] Authorisation no. 9/2014—General Authorisation to Process Personal Data for Scientific Research Purposes. https://www.gazzettaufficiale.it/atto/serie_generale/caricaDettaglioAtto/originario?atto.dataPbblicazioneGazzetta=2014-1230&atto.codiceRedazionale=14A09916&elenco30giorni=true.

[25] Law Decree 22 December 2017, No. 219, published in the Ocial Gazette No. 12 of 16 January 2018. https://www.gazzettaufficiale.it/eli/gu/2018/01/16/12/sg/pdf.

[26] Traggiai E., Becker S., Subbarao K., Kolesnikova L., Uematsu Y., Gismondo M.R., Murphy B.R., Rappuoli R., Lanzavecchia A. (2004). An efficient method to make human monoclonal antibodies from memory B cells: Potent neutralization of SARS coronavirus. Nat. Med..

[27] Hilpert K., Winkler D.F., Hancock R.E. (2007). Peptide arrays on cellulose support: SPOT synthesis, a time and cost-efficient method for synthesis of large numbers of peptides in a parallel and addressable fashion. Nat. Protoc..

[28] Winkler D.F., Campbell W.D. (2008). The spot technique: Synthesis and screening of peptide macroarrays on cellulose membranes. Methods. Mol. Biol..

[29] Lin M., Mcrae H., Dan H., Tangorra E., Laverdiere A., Pasick J. (2010). High-resolution epitope mapping for monoclonal antibodies to the structural protein Erns of classical swine fever virus using peptide array and random peptide phage display approaches. J. Gen. Virol..

[30] Di Bonito P., Nicoletti L., Mochi S., Accardi L., Marchi A., Giorgi C. (1999). Immunological characterization of Toscana virus proteins. Arch. Virol..

[31] Backovic M., Johansson D.X., Klupp B.G., Mettenleiter T.C., Persson M.A., Rey F.A. (2010). Efficient method for production of high yields of Fab fragments in Drosophila S2 cells. Protein Eng. Des. Sel..

[32] Reed L.J., Muench H. (1938). A simple method of estimating fifty per cent endpoints. Am. J. Hyg..

[33] Hlavacek W.S., Posner R.G., Perelson A.S. (1999). Steric effects on multivalent ligand-receptor binding: Exclusion of ligand sites by bound cell surface receptors. Biophys. J..

[34] Rizk R.Z., Christensen N.D., Michael K.M., Müller M., Sehr P., Waterboer T., Pawlita M. (2008). Reactivity pattern of 92 monoclonal antibodies with 15 human papillomavirus types. J. Gen. Virol..

[35] Elliott R.M., Brennan B. (2014). Emerging phleboviruses. Curr. Opin. Virol..

